# Development and validation of a new anthropometric equation to predict fat mass percentage in a heterogeneous Caucasian population

**DOI:** 10.1017/S136898002400209X

**Published:** 2024-10-22

**Authors:** Daniel Rojano-Ortega, Heliodoro Moya-Amaya, Antonio Molina-López, Antonio Jesús Berral-Aguilar, Francisco José Berral-de la Rosa

**Affiliations:** 1 CTS-595 Research Group, Department of Informatics and Sports, Universidad Pablo de Olavide, Sevilla 41013, Spain; 2 Department of Nutrition of Udinese Calcio, Udine, Italy

**Keywords:** Body composition, Skinfolds, Kinanthropometry, Adipose tissue

## Abstract

**Objective:**

(1) To develop a new regression equation for estimating fat mass percentage (%FM) from anthropometric measurements in a heterogeneous Caucasian population and (2) to compare it with the Durnin and Womersley equation, which is one of the most used anthropometric equations for FM assessment.

**Design:**

Body mass, stature and four skinfolds (biceps, triceps, subscapular and supracrestal) were assessed by an accredited anthropometrist, according to the International Society for Advancement in Kinanthropometry. Participants completed a dual-energy X-ray absorptiometry (DXA) whole-body scan to determine their %FM. A new anthropometric equation to estimate %FM was developed using multiple forward regression analyses with DXA as the reference method. Tests for the accuracy of the different equations included mean differences, coefficient of determination, SE of the estimate (SEE), concordance correlation coefficient (CCC) and Bland–Altman plots.

**Setting:**

Spain.

**Participants:**

Two hundred and eighteen healthy Caucasian participants aged 18–65 years participated in this cross-sectional study.

**Results:**

Our proposed equation explained 89·9 % of the variance in the DXA-derived %FM, with a low random error (SEE = 3·00 %), a very strong agreement (CCC = 0·93), no fixed or proportional bias and a relatively low individual variability (5·84 %). However, the Durnin and Womersley equations obtained a fixed bias of –3·65 % when compared with DXA and a greater individual variability (6·74 %).

**Conclusions:**

The proposed equation can accurately estimate %FM in a heterogeneous Caucasian population with a wide age range (18–65 years). Additionally, the Durnin and Womersley equation was inadequate when applied to our participants.

Body composition describes the amounts of the various components of the human body^(1)^. It plays an important role in multiple areas. For instance, fat mass (FM) has traditionally been used to monitor training effects in athletes because, in most sport modalities, excess FM reduces performance^(2,3)^. FM has also been related to health status in non-athletes due to its association with various pathologies, such as cardiovascular and metabolic disease^(4,5)^. In addition, inadequate levels of skeletal muscle mass or bone mineral content have also been associated with an increased injury risk in the elderly^(6)^.

Therefore, body composition assessment is essential to monitor health status. However, directly assessing of body composition ‘in vivo’ is impossible^(1)^, and several indirect or double-indirect methods have appeared over time, such as skinfold thicknesses, bioelectrical impedance or dual-energy X-ray absorptiometry (DXA)^(7)^. Indirect methods use assumptions or algorithms to estimate a certain body composition component, while double-indirect methods use regression equations that have been validated from indirect methods^(8)^.

Some indirect methods have been considered reference methods for different body composition parameters^(9)^. However, these methods generally require expensive instruments and competent technical expertise and are difficult to implement in large population-based studies^(8)^. Therefore, many studies have used bioelectrical impedance or surface anthropometry to estimate body composition, despite their limited accuracy^(8,10)^.

Surface anthropometry is one of the most widely used methods for estimating body composition because it is inexpensive, easy to use and transport and provides relatively quick results^(11,12)^. Furthermore, it has shown sufficient validity and reliability in assessing body composition^(13,14)^. Body composition can be estimated from anthropometric measurements at two different levels: the molecular level, which fractionates the body into lipid and lipid-free masses, and the anatomical or tissue level, which fractionates the body mass into adipose tissue, skeletal muscle, bone and lean soft tissue masses^(15,16)^. Anthropometric parameters are introduced into specific equations to determine body composition at one of these two levels.

The first strategy to fractionate the body mass into its anatomical components using anthropometric measurements was developed by Matiegka^(17)^. Many researchers have since proposed different equations for estimating the different body compartments. Except from a few formulas validated with cadaveric dissection^(16)^, most of them have been validated using indirect methods considered as the gold standard.

As mentioned above, assessing FM has become important in clinical practice because, even if different patterns of adiposity have been observed across different ethnic groups^(18)^, higher amounts of FM have been associated with cardiovascular risk factors and elevated plasma glucose levels^(19,20)^, which may predict the development of CVD and type 2 diabetes. In addition, obesity is the accumulation of FM to the extent that it may have adverse effects,^(21)^ and its prevalence has considerably increased worldwide over the last decades^(22)^. The overall energetic caloric content of the diet due to the changes in the quantity and the quality of the food consumed, together with the drop of daily physical activity have created an ‘obesogenic environment’^(23)^.

One of the main problems when comparing FM estimations using different methods is the confusion between lipid mass and adipose tissue mass, which have been used interchangeably despite not being equivalent^(16)^. Lipid mass (TAG) is composed of lipids and glycerol, while adipose tissue refers to the total mass of the adipose cells (adipocytes), which have a high TAG content but are also composed of water and minerals^(24)^.

Skinfold thickness is used to estimate FM based on the relationship between subcutaneous adipose tissue and total body fat^(25)^. Some skinfolds and other anthropometric parameters are introduced into one anthropometric equation to estimate FM. Except for the Ross and Kerr formula^(26)^, the rest of the traditional anthropometric formulae estimate lipid mass^(16)^. Many researchers have developed new regression equations for estimating FM in adults without further requirements regarding age, BMI, stature or physical activity level^(27–29)^. However, these new equations have not prevailed, likely because they have not achieved great accuracy in estimating body fat. The reality is that, for general populations, old traditional equations continue to be the most used ones, despite being shown to underestimate or overestimate FM^(27–30)^.

In fact, according to a review performed by Marin-Jimenez et al.^(31)^, who analysed the validity of equations for body composition in adults, the Durnin and Womersley equation, which uses only the sum of four skinfolds, was the most used in the included studies. However, in addition to having demonstrated poor accuracy for estimating FM in different populations, the Durnin and Womersley equation estimates lipid mass and not adipose tissue mass, and it is the latter that has shown a relationship with health or disease in most recent studies^(32–34)^.

Because of this strong positive relationship between FM and the risk of chronic diseases and because assessing FM is also important in the control of the current obesity epidemic, a more accurate equation for estimating FM across different age or BMI ranges might enhance the early detection and diagnosis of some health problems and help nutrition specialists monitor progression during dietary interventions.

Therefore, this study aimed to develop a new regression equation for estimating FM percentage (%FM) from anthropometric measurements in a heterogeneous Caucasian population and validate it with DXA as the ‘criterion standard’. We also aimed to compare the new equation with the Durnin and Womersley equation for males aged 17–72 years and females aged 16–68 years^(35)^.

## Methods

### Participants

This study recruited 218 healthy Caucasian participants aged 18–65 years from Spanish populations living in Andalusia, with no requirements for BMI or physical activity level. However, due to some errors in the DXA scan, mainly caused by patient movement during the measurement process, only 206 participants were included in subsequent analyses. Participants were contacted via email through different Andalusian associations and universities.

### Experimental design

Participants were randomly assigned to one of two groups using Microsoft Excel’s randomise function: a development group (*n* 141, ∼70 %) and a validation group (*n* 65, ∼30 %). One prediction equation was developed with the data from the development group and then cross-validated on the validation group. Due to the relatively balanced distribution of our participants in terms of sex, age range, BMI and level of physical activity, both groups were considered as representative of a general Caucasian population (Table 1). A minimum sample size of 103 participants was determined (power = 0·8, *α* = 0·05; G * Power v. 3.1.9.7, Dusseldorf, Germany) to achieve a medium effect size for the coefficient of determination (R^2^) increases, in a regression equation with up to seven predictors. Therefore, our sample size of 141 participants was enough to ensure an adequate power analysis in the equation development.

**Table 1.** Sex, age range BMI and physical activity distribution of the development and validation groups

	Development (*n* 141)	Validation (*n* 65)
Sex		
Women	70	30
Men	71	35
Age range		
< 30 years	38	18
30–50 years	52	23
> 50 years	51	24
BMI		
< 20	11	7
20–25	63	28
25–30	47	20
> 30	20	10
Physical activity		
–	44	19
1–2 d/week	52	26
> 2 d/week	45	20

All participants underwent body composition assessment via DXA and surface anthropometry to develop and validate the new %FM anthropometric prediction equation against a reference method (DXA) and to compare it with the Durnin and Womersley equation. All measurements were taken on the same morning with an empty bladder, either after an overnight fasting or at least 3 h after a light breakfast. The participants were instructed to refrain from vigorous exercise and the consumption of alcohol or any stimulant substance at least 12 h before the evaluation.

### Dual-energy X-ray absorptiometry

Immediately after anthropometric measurements, the same technician performed a whole-body QDR Series Horizon DXA scan (Software version Apex 5.6.1.3; Hologic Inc.) using the manufacturer’s recommended procedures. The DXA scanner was calibrated daily according to the manufacturer’s instructions. All the scans were performed in a ventilated room at the same temperature. Participants were instructed to remove all metal objects and to remain motionless in a supine position with their arms extended by their sides. After the scans, bone mineral content, %FM (adipose tissue mass) and percentage of fat-free mass were obtained. The within-subject CV for %FM was measured previously in our laboratory with 25 participants was 0·9 %.

### Anthropometric measurements

Anthropometric measurements were performed according to the International Society for Advancement in Kinanthropometry^(36)^ by the same anthropometrist accredited by the Andalusian Medical Association for Physical Education and Sport, which follows International Society for Advancement in Kinanthropometry guidelines. All equipment was calibrated as recommended by the manufacturers. Body height was measured to the nearest 0·1 cm with a stadiometer (SECA) with participants wearing light clothing. Body weight was measured to the nearest 0·1 kg with a body composition analyser (Tanita). The four skinfolds (biceps, triceps, subscapular and supracrestal) were measured to the nearest 0·5 mm using a calliper (Slim Guide, Rosscraft) with participants standing in anatomical position. Skindfolds were measured in duplicate and the mean was used for all analysis. The technical error of measurement calculated with twenty-five participants of the study ranged from 2·36 % to 4·15 %, which is considered as acceptable for a skillful anthropometrist^(37)^.

### Statistical analysis

Statistical analyses were performed using SPSS for Windows (v. 22.0; SPSS Inc.). The means and standard deviations of all variables were calculated. All variables were first tested for normality using Kolmogorov–Smirnov tests. As this condition was met, unpaired sample Student’s *t* tests were used to determine significant differences between mean values of the development and validation groups.

Multiple forward regression analyses were performed to develop the new %FM equation with DXA %FM as the dependent variable. The use of the whole sample produced better results than stratified regression analyses by age, sex or BMI. Therefore, a single equation for the whole sample was developed with anthropometric variables, age and sex as independent variables. Significance was set at *P* < 0·05 for inclusion and at *P* > 0·1 for removal. Normality and homoscedasticity of the residuals in the new model were confirmed and the Durbin–Watson value was considered acceptable (1·5–2·5). Multicollinearity was tested using the variance inflation factor (VIF). Any variable with a VIF > 10 was considered redundant and eliminated^(38)^. To cross-validate the Durnin and Womersley equation and the new developed equation, the estimated %FM calculated with both equations in the validation group, was first tested for normality using Kolmogorov–Smirnov tests. As this condition was met, paired sample Student’s t-tests were conducted to assess differences between DXA-derived %FM and equations-predicted %FM. Then, coefficients of determination (R^2^) and se of the estimate were calculated. Concordance correlation coefficients (CCC) were also calculated using Lin’s methodology^(39)^. The following cut-off points were used for CCC: negligible concordance (0·00–0·09); weak concordance (0·10–0·39); moderate concordance (0·40–0·69); strong concordance (0·70–0·89) and very strong concordance (0·90–1·00)^(40)^. Finally, Bland–Altman graphs were created for the proposed equation and the Durnin and Womersley equation to assess their fixed bias (mean difference significantly different from 0), the 95 % limits of agreement (LoA) and to identify whether proportional bias was present (trends). Statistical significance was set at *P* < 0·05.

## Results

The descriptive characteristics of the development and validation groups are presented in Table 2. No significant differences were found between groups. Since first regression analysis in the development group found that stature, body mass and BMI had a VIF > 10, only two of them were included in subsequent regressions to avoid collinearity. The best equation finally included triceps, subscapular and supracrestal skinfolds; sex; age; stature and body mass (Table 3) and explained 88·6 % of the variance (R^2^ = 0·886) with an se of the estimate of 2·79 %. The selected prediction equation was as follows for both males (sex = 1) and females (sex = 0):

**Table 2.** Descriptive characteristics of development and validation groups

Variables	Development (*n* 141)		Validation (*n* 65)	
	Mean	sd	Mean	sd
Age (years)	42·78	14·03	41·48	14·94
Body mass (kg)	72·91	13·08	73·72	11·69
Stature (cm)	168·66	9·53	169·70	9·25
BMI (kg/m^2^)	25·57	3·58	25·68	3·74
Skinfolds				
Triceps (mm)	13·30	5·83	12·99	6·61
Biceps (mm)	6·89	4·21	6·84	4·45
Subescapular (mm)	16·72	7·76	15·48	7·94
Supracrestal (mm)	22·84	7·91	22·33	8·95
DXA FM (%)	30·47	8·22	28·81	9·36

DXA, dual energy X-ray absorptiometry; FM, fat mass.

**Table 3.** Regression analysis for the development of the new predictive equation

Variables	Coefficient	R^2^ change	*P* value	VIF
Constant	48·645	–	–	–
Triceps Sk.	0·159	0·667	0·000	3·736
Sex	−8·808	0·084	0·000	4·005
Subescapular Sk.	0·210	0·081	0·000	2·945
Supracrestal Sk.	0·177	0·019	0·000	1·972
Age	0·070	0·018	0·000	1·283
Body mass	0·156	0·011	0·0162	5·563
Stature	−0·224	0·006	0·000	4·200
Model	–	0·886	0·000	–

Sk, skinfold; R^2^, coefficient of determination; VIF, variance inflation factor.

%FM = 48·645 + 0·159·triceps – 8·808·sex + 0·210·subescapular + 0·177·supracrestal + 0·070·age + 0·156·body mass – 0·224·stature

where skinfolds are in mm, age in years, body mass in kg and stature in cm.

**Figure 1.**
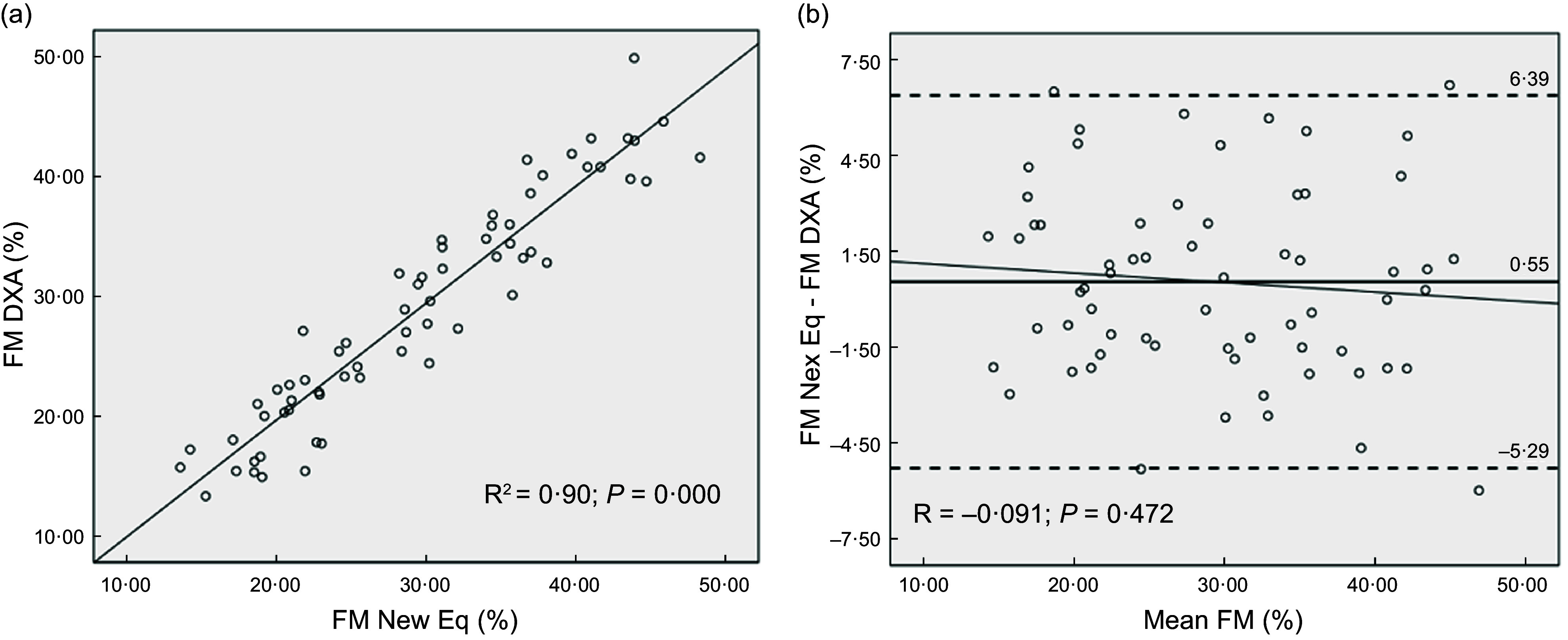
Fat mass percentage predicted with the new equation *v.* DXA-derived fat mass percentage in the validation group (a). Bland–Altman plots showing the fixed bias and 95 % limits of agreement (±1·96 sd) between the fat mass percentages differences and means of DXA and the new equation in the validation group (b). The correlation between de difference of the methods and the mean of the methods (trends) are also shown (b). DXA, dual-energy X-ray absorptiometry.

**Figure 2.**
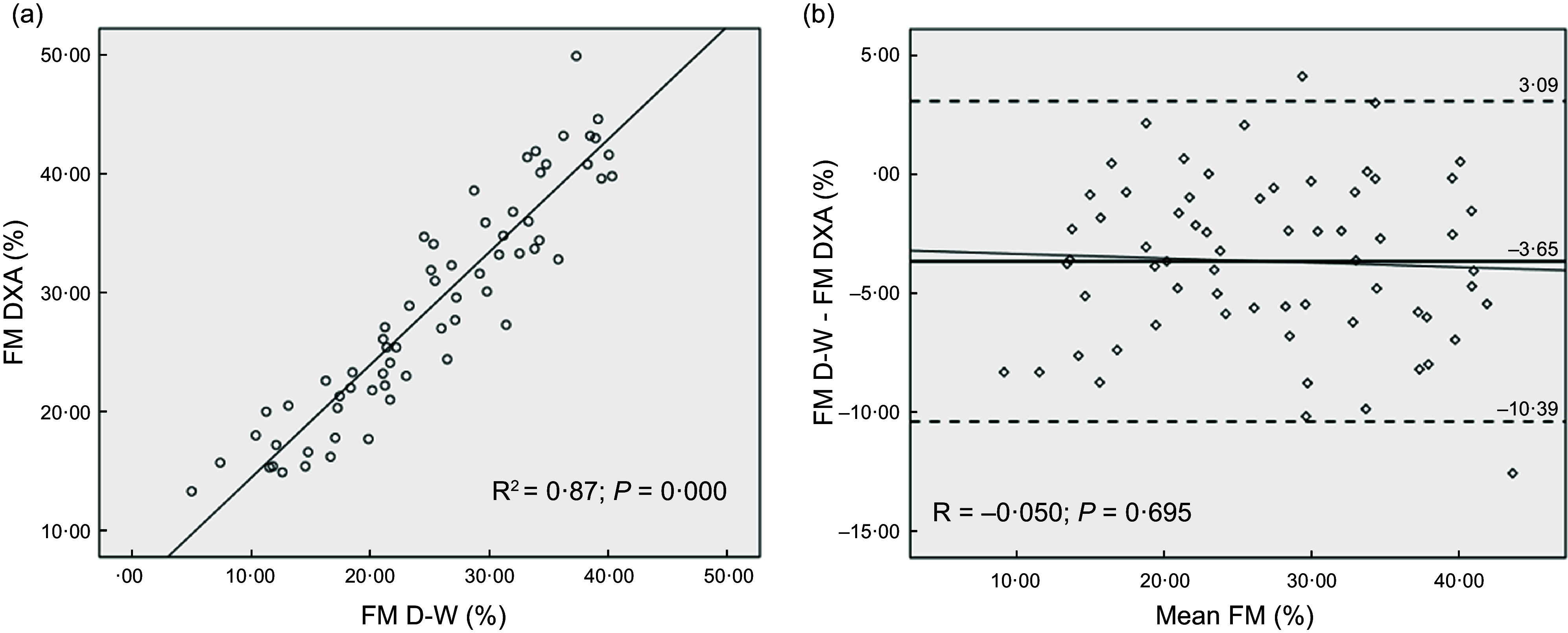
Fat mass percentage predicted with the Durnin and Womersley equation *v.* DXA-derived fat mass percentage in the validation group (a). Bland–Altman plots showing the fixed bias and 95 % limits of agreement (±1·96 sd) between the fat mass percentages differences and means of DXA and the Durnin and Womersley equation in the validation group (b). The correlation between de difference of the methods and the mean of the methods (trends) are also shown (b) DXA, dual-energy X-ray absorptiometry.

Table 4 shows the cross-validation analysis of the new prediction equation and the Durnin and Womersley equation in the validation group. There was no significant difference in the mean %FM values of the sixty-five participants between DXA and the new equation. However, the Durnin and Womersley equation underestimated mean %FM compared with DXA. Coefficient of determination for the new equation was higher, accounting for 89·9 % of the variance and the se of the estimate was lower. In addition, Lin’s CCC indicated strong agreement with DXA for the Durnin and Womersley equation (CCC = 0·85) and very strong agreement for the new equation (CCC = 0·93). The least square regression between DXA-derived %FM and the predicted %FM is presented in Fig. 1(a) for the new equation and in Fig. 2(a) for the Durnin and Womersley equation.

**Table 4.** Validation via DXA of anthropometric equations for fat mass percentage prediction

	FM %		R^2^	*P*	SEE (%)	CCC (Lin)
	Mean	sd				
New Equation	29·36	9·09	0·899	*P* < 0·001	3·00	0·93
Durnin and Womersley	25·16	9·19	0·867	*P* < 0·001	3·44	0·85

DXA, dual-energy X-ray absorptiometry; FM, fat mass; R^2^, coefficient of determination; SEE, standard error of the estimate; CCC, concordance correlation coefficient.

The accuracy of both equations was further assessed with Bland–Altman plots (Fig. 1(b) and Fig. 2(b)). The Bland–Altman analyses showed no fixed bias between the new equation and DXA. However, the Durnin and Womersley equation showed a fixed bias of –3·65 % and had a broader 95 % LoA (–10·39 %, 3·09 %). None of the equations showed significant trends between the differences and the means (*P* > 0·05).

## Discussion

This study developed a new %FM prediction equation for a heterogeneous Caucasian population and compared it with the Durnin and Womersley equation, one of the most used equations for %FM prediction in a general population with various body types ages (16–72 years)^(16,29,31)^. DXA was used as the reference method.

Only a few studies have tried to validate the Durnin and Womersley equation in populations with a broad age range, concluding that it failed to show good accuracy^(28,29)^. Peterson et al.^(29)^ validated the Durnin and Womersley equation for %FM prediction in a group of white males and females aged 18–55·6 years, using a four-compartment model as the reference method. They obtained a mean underestimation of –2·8 % in males and –1·8 % in females, and the 95 % LoAs were of about 10 %. Hastuti et al.^(28)^ used the deuterium dilution technique to validate the Durnin and Womersley equation in a group of Indonesian males aged 18–65 years, and they obtained a fixed bias of –0·8 % and a 95 % LoA of 7·7 %.

Their results agree with ours, even in populations with different ethnicities and BMI. In our validation group, the Durnin and Womersley equation showed a fixed bias of –3·65 %, which confirmed that this equation would not be valid for estimating the %FM of the whole group. This greater underestimation is likely due to two different reasons: First, an overestimation of body density because a small overestimation of it can result in a considerable underestimation of %FM^(29)^; second, to the fact that the Durnin and Womersley equation estimates lipid mass, which is always lower than adipose tissue mass^(24)^. In addition, a 95 % LoA of 6·74 % indicated that 95 % of individuals’ predicted %FM could be up to ± 6·74 % above or below their reference value. Therefore, according to the previous and present investigations, the Durnin and Womersley equation cannot be considered adequate at the group or individual level.

Many studies have also demonstrated the poor accuracy of the Durnin and Womersley equation for predicting %FM in specific populations with a more restrictive age range. Some have also proposed and validated new equations with better results^(38,41,42)^. However, none of these new equations have prevailed, and traditional equations continue to be widely used, including the Durnin and Womersley equation. The failure of these new equations is likely due to two important reasons: (1) The equations with good results, both at the group and individual levels, were developed for specific populations with over-restrictive conditions and/or small sample sizes^(41,42)^ and (2) those equations developed for general populations obtained good results at the group level but not at the individual level, obtaining a 95 % LoA broader than 7·5 %^(28,29)^.

Because we only measured biceps, triceps, subscapular and supracrestal skinfolds, comparing our new equation with other traditional anthropometric equations, such as the Jackson and Pollock equation, was not possible. However, some studies have attempted to validate this equation against %FM, calculated with hydrodensitometry or a four-compartment model, in two groups of white females with wide age and BMI ranges, finding that the Jackson and Pollock equation underestimated %FM compared with the reference methods^(27,29)^.

Estimating FM from skinfolds is widely used in both clinical and epidemiological settings due to its methodological simplicity, low cost and non-invasive nature^(43)^. However, traditional equations measure lipid mass and not adipose tissue mass and have repeatedly demonstrated poor accuracy. Our proposed equation estimates adipose tissue mass and uses sex, age, stature and body mass as independent variables, making them valid for a wider population. In addition, it also includes three skinfolds, and in the validation group it explained 89·9 % of the variance in the DXA-derived %FM, with a low random error (se of the estimate = 3·00 %), a very strong agreement (CCC = 0·93), no fixed or proportional bias and a relatively low individual variability (5·84 %). These better results are likely due to the inclusion of a higher number of variables, which is not an inconvenient because any accredited anthropometrist can easily and quickly take those measures.

Assessing nutritional status is essential in both clinical practice and research, and body composition provides valuable information, helping detect several important nutritional problems. Anthropometry has been widely used as an effective tool to estimate body composition. However, new, more accurate equations in different populations are needed, particularly to estimate FM, because higher amounts of FM have been associated with cardiovascular risk factors and may predict the development of other diseases in at-risk populations. Since our new equation explains a large proportion of the variability in the DXA-derived %FM, has a very strong CCC, shows no bias and a sufficiently narrow 95 % LoA, we encourage its use to estimate %FM percentage in general Caucasian populations.

As Heymsfield and Strauss^(44)^ affirm, the use of internet technology and artificial intelligence will certainly improve body composition prediction models, when clinical investigators all over the world upload together their data to a specially designed cloud storage site. Until that time comes, the development of equations like ours may be extremely useful.

Despite the positive results obtained in the present investigation, some limitations must be acknowledged. First, the reliability of our new equation remains to be measured. Second, even if our equation estimates %FM better than the Durnin and Womersley equation, it may not be a better predictor of the different pathologies associated with high amounts of FM. Third, while most recent studies use DXA as the reference method, a four-compartment approach would have been better because it is considered the true reference method for assessing body composition.

Future studies are recommended to validate the new equation or develop new similar ones in other populations with different ethnicities. In addition, since all our participants lived in a restricted region of Spain, new equations may have to be developed for Caucasians from different geographical areas. Further investigations should also develop %FM prediction equations in specific populations, using the independent variables included here, which are likely the basis of our good results, both at the group and individual levels.

### Conclusion

The strong positive relationship between FM and risks for some chronic diseases and the increasing prevalence of obesity with its adverse health consequences demand more accurate anthropometric equations for estimating FM across different age and BMI ranges. The regression equation developed in this study for estimating %FM using DXA as the criterion method can accurately estimate %FM in a heterogeneous Caucasian population with a broad age range (18–65 years). However, when applied to our participants, the Durnin and Womersley equation was inappropriate. Our new equation explains a large proportion of the variability in the DXA-derived %FM, has a very strong agreement, shows no fixed or proportional bias and a relatively narrow 95 % LoA. These promising results are likely due to the inclusion of a high number of variables, including sex, age, stature, body mass and three skinfolds, all of which can be measured easily and quickly by any accredited anthropometrist. This accurate equation for estimating %FM in a heterogeneous Caucasian population can enhance the early detection and diagnosis of some health problems and nutrition specialists may find it very useful for monitoring progression during dietary interventions.
